# Ehlers Danlos Syndrome: An Unusual Presentation You Need to Know about

**DOI:** 10.1155/2013/764659

**Published:** 2013-05-16

**Authors:** Amel Karaa, Joan M. Stoler

**Affiliations:** ^1^Boston Children's Hospital, Harvard Medical School, Boston, MA 02115-5724, USA; ^2^Boston Children's Hospital, Genetics Division, Hunnewell 536, 300 Longwood Avenue, Boston, MA 02115, USA

## Abstract

The Ehlers Danlos syndromes (EDS) comprise a group of connective tissue disorders characterized by tissue fragility of the skin, ligaments, blood vessels and internal organs. Variable degrees of clinical severity and organ involvement are due to the molecular and biochemical heterogeneity of this group of disorders and have led to classification into well-characterized subtypes that are extending with the discovery of new genes and overlapping syndrome. Types include classical EDS (EDS I/II), hypermobility EDS (EDS III), vascular EDS (EDS IV), kyphoscoliosis EDS (EDS VI), arthrochalasia (EDS VIIA, B) and Dermatospraxis (EDS VIIC). Even to the well trained professional, the diagnosis of EDS remains a challenge due to overlapping symptoms and cases can remain without a well-defined classification. Life altering complications of this group of disorders include vascular and hollow organ rupture and ligamentous laxity leading to chronic dislocation with ensuing pain and long term disability. Patients initially present to the general practitioner who is expected to recognize the symptoms of EDS and to proceed with appropriate referral for definitive diagnosis and management to prevent devastating complications. In this paper, we describe a male with classical EDS complicated by devastating vascular and orthopedic events.

## 1. Introduction 

Ehlers-Danlos syndrome (EDS) consists of a heterogeneous group of disorders which are part of a larger group of connective tissue disorders (also including Marfan and Loeys-Dietz syndromes). The Ehlers-Danlos syndromes are characterized by abnormalities of the connective tissue of the skin, ligaments, blood vessels, and internal organs leading to ligamentous laxity and variable skin and tissue fragility. The diverse molecular and biochemical background lead to variable degrees of tissue and organ involvement, accounting for the EDS subgroups and overlapping phenotypes (Table 1). The prevalence of EDS is estimated at 1 in 5000 live births, and some suggest a higher prevalence as physicians' awareness of the disease improves [1]. Some but not all of the types can be confirmed by genetic testing after clinical criteria are met. However, even to the well-trained professional, many cases remain ambiguous and do not fit in any of the well-described subtypes. 

The different EDS disorders are classified according to the joint and skin involvement. Individuals with classical EDS (EDS I/II) have increased skin extensibility, difficulty with skin healing, atrophic scars, easy bruisability, lax joints, and recurrent dislocations. Individuals with hypermobility EDS (EDS III) have normal wound healing, recurrent joint dislocations, joint pain, and joint laxity. The features of vascular EDS (EDS IV) include thin, fragile translucent skin, atrophic scars, easy bruisability, increased risk for pneumothorax, and spontaneous organ and vascular rupture. The features of kyphoscoliosis EDS (EDS VI) are significant hypotonia, progressive early-onset scoliosis, lax joints, poor wound healing, atrophic scars, and risk for eye globe and vascular rupture. Individuals with arthrochalasia (EDS VIIA, EDS VIIB) are born with hip dislocations and have significant joint dislocations, joint hypermobility, and increased risk of fractures. EDS VIIC (dermatosparaxis EDS) consists of quite lax skin, poor wound healing, and atrophic scars. In the last years, new molecular insights and gene discovery have permitted the expansion of the EDS spectrum to several new subtypes with overlapping manifestations prompting some to consider refining current nosological classification [2] (Table 1). For example, different mutations in the *COL1A1/COL1A2 *genes (which code for pro-*α*1 (I) and pro-*α*2 (I) chains, resp.; both components are of type I collagen, the most abundant form of collagen in the body), can result in a phenotype of classical type EDS, arthrochalasia type EDS, mild to severe osteogenesis imperfecta (OI) or an overlapping phenotype of EDS/OI resulting in symptoms of EDS, joint hypermobility, skin hyperextensibility, atrophic scarring, and easy bruising in association with features of osteogenesis imperfecta, bone fragility, short stature, and blue sclerae. 

In this paper, we describe a patient with an unusual presentation, a male with classical EDS complicated by devastating vascular and orthopedic events.

## 2. Case Report

Our patient was seen for evaluation for possible EDS at the age of 33 months due to increased bruising and a history of skin “splitting” with minor trauma. He was the product of a pregnancy complicated by an ultrasound suggestive of hydronephrosis which later resolved and maternal preeclampsia requiring hospitalization and rupture of membranes at 33 weeks. Delivery was by caesarean section for breech presentation, and he remained in the NICU for three weeks for growth and feeding concerns. He experienced significant bruising with minor trauma, including at the age of 18 months a fall from a bed which resulted in a large forehead hematoma and an ankle laceration requiring 32 stitches, and he was diagnosed with mild Von Willebrand disease. Developmental milestones were all age appropriate. Family history was noncontributory. He was diagnosed with classical EDS (type II) at 4 years old on the basis of clinical findings of soft, doughy, and slightly hyperextensible skin, slightly increased range of motion of the joints, and easy bruising with skin fragility. He had no major health concerns until the age of 11 years old when he acutely presented with abdominal pain. He was found to have a superior mesenteric artery aneurysm with thrombosis initially treated with anticoagulation. At that time examination revealed soft and hyperextensible skin, atrophic scars (on shin and ankles), high palate, significant pectus excavatum, kyphoscoliosis, long fingers, long toes, and hypermobile joints. 

**Figure 1:**
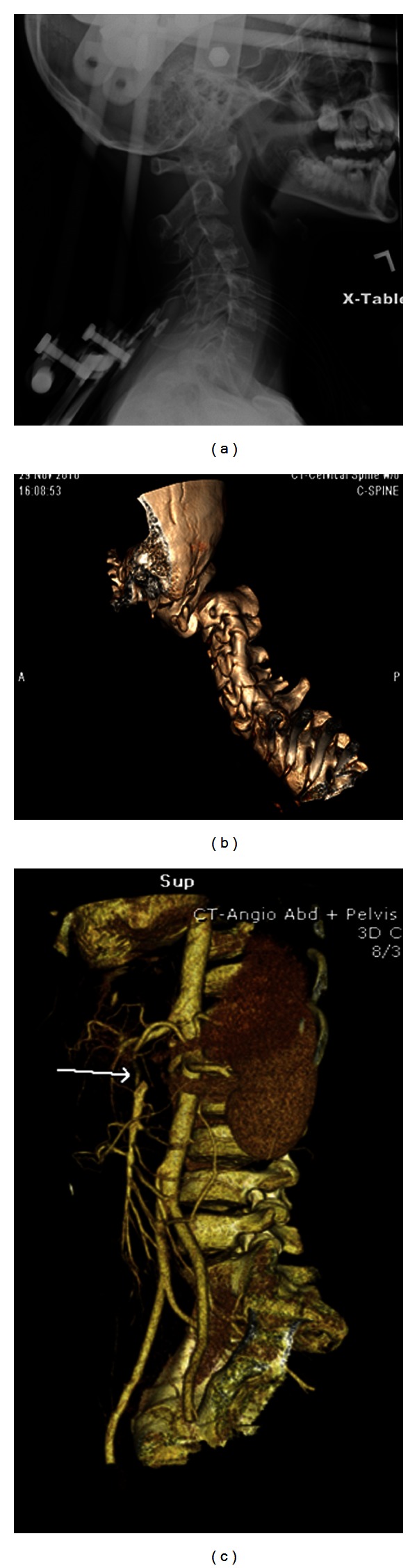
Lateral view (a) and 3D CT reconstruction (b) of the cervical spine of the patient. Note the severe posterior C1/C2 subluxation with posterior displacement of the C2 vertebral body in relation to C1. (c) 3D reconstruction from a CT angiogram for the patient, showing the filling defect of the inferior mesenteric artery (arrow).

 A week later, he experienced more severe abdominal pain and hypotension secondary to rupture of the inferior mesenteric artery with massive hemorrhaging (Figure 1(c)). These led to multiple procedures including coiling and in situ thrombolysis of the aneurysms, a right hemicolectomy, cholecystectomy, and small bowel resection of the distal ileum with end-ileostomy as a result of intestinal gangrene. He was also found to have a bilateral subclavian artery thrombosis and an infarction of part of his liver. As there were multiple collateral vessels observed during surgery, it was thought that the aneurysms, especially of the mesenteric artery, were longstanding. He was admitted 3 months later for the evaluation of neck pain and was found to have critical C1-C2 cervical spine instability requiring emergency cervical spine fusion (Figures 1(a) and 1(b)). The following month, he had recurrence of abdominal pain and syncope due to an aneurysm of the celiac artery with dissection requiring coiling of the aneurysm. 

Testing of the *COL5A1* gene confirmed the diagnosis of classical EDS revealing a missense mutation in exon 34, c.2765G>A. Biochemical analysis of types I and III procollagen was within normal limits. Evaluation for EDS IV, EDS VI, and Marfan and Loeys-Dietz syndrome involved sequencing and deletion/duplication analysis of the *COL3A1, TGF*β*R1*, *TGF*β*R2,*  and  *FBN1* genes as well as deoxypyridinoline/pyridinoline ratio in the urine, all of which were normal.

Despite the severity of his presentation, the patient is now 14 years old and only suffering from increased thoracolumbar and core weakness managed by physical therapy. 

## 3. Discussion

Arterial ruptures and sudden death complications of Ehlers-Danlos syndrome were recognized by Mories in the 1960s on postmortem examinations [3]. Barabas was the first in 1967 [4] to classify EDS into subtypes and to recognize vascular EDS as a lethal entity with “liability to gross bruising, the peculiar transparency of the skin, the minor degrees of skin hyperextensibility and joint hypermobility, the attacks of severe abdominal pain, and an impending arterial catastrophe” [4]. Forty-five years later, we still recognize arterial rupture as the hallmark of vascular EDS (EDS IV) with intestinal and organ perforation which we regard as specific feature to this subtype caused by a *COL3A1* gene mutation. Indeed, 80% of vascular EDS patients have been shown to have a life-threatening complication by the age of 40 [5]. Although the phenotypic variability in EDS is challenging, we make it a mission not to miss diagnosis given the implications for the patients' life and for familial genetic counseling. 

Very rare reports of vascular complications occurring in classical EDS have been published. These included a right iliac artery dissection in a 37-year-old female, a left femoral and aortic aneurysm dissection in a 42-year-old man and a dissection of the infrarenal aorta and left iliac artery in a 39-year-old individual. All three probands had classical-like EDS with *COL1A1* mutations [6]. A fourth case describing a 42-year-old man with classical EDS (due to a mutation in COL5A1) with rupture of the left common iliac artery has also been published [7]. These authors argued that other genetic and environmental factors such as hypertension (in older patients) could have contributed to the *COL5A1* already challenged vessel wall [7]. 

Joint dislocation is a common symptom in EDS. It usually affects limb joints (shoulders, hips, knees, and fingers) and can occur with minimal or no trauma. Vertebral dislocation; however, is less recognized and uncommonly reported. In a study by Halko et al. [8], twenty-six asymptomatic patients with different EDS subtypes attending a national meeting were selected and radiographed with lateral extension-flexion radiographs and were found to have evidence of atlantoaxial subluxation in 2 patients, “horizontal translation” of C2 in 3 patients and cervical arthrosis in 9 patients. It very well may be that this is an underrecognized complication in EDS. 

We here present the youngest case of classical EDS with severe arterial dissections and who had significant cervical instability requiring immediate stabilization. This case has important clinical implication for counseling and management of individuals with EDS. In cases with arterial rupture and no *COL3A1*, *TGF*β*1, TGF*β*2,* and *FBN1* gene mutations, attention to signs of classical EDS and analysis of the *COL5A1, COL5A2,* and* COL1A1* genes may be justified. Patients with classical EDS should be appropriately counseled for risks of arterial rupture, cervical spine instability, and avoidance of high impact activities and should seek immediate medical attention in case of any unusual acute symptoms. 

In summary, we present rare manifestations of EDS such as vascular rupture and cervical spine dislocation in a patient with classical EDS. Although uncommon, physicians need to be made aware of the implications of such findings in order to refer the patients for appropriate diagnosis, family counseling, and, more importantly, for a thorough evaluation and screening of life-threatening and devastating vascular and spinal abnormalities that can potentially be prevented.

## Figures and Tables

**Table 1:** Overview of Ehlers-Danlos syndromes (adapted from De Paepe and Malfait [2].)

EDS subtypes (former type)	Inheritance	Major symptoms	Genes
Classic (I/II)	AD	Skin hyperextensibility Widened atrophic scars	*COL5A1/COL5A2* *COL1A1*
	AR	Joint hypermobility, muscle weakness, and distal contractures	*TNX-B *
Hypermobility (III)	AD	Generalized hypermobility and subtle skin findings	*? *
Vascular (IV)	AD	Arterial and hollow organ rupture at a young age	*COL3A1 *
Vascular-like	AD	Features of both classic and vascular types	*COL1A1 *
Cardiac-valvular	AR	In childhood: mild skin, joint hypermobility, hypotonia, and osteopenia. In adulthood: severe valve disease	*COL1A2 *
EDS with periventricular heterotopia	XLR	Nodular brain heterotopia and classic EDS symptoms	*FLNA* *ARFGEF2 *
Kyphoscoliotic (VIa)	AR	Early progressive kyphoscoliosis	*PLOD1 *
Musculocontractural (VIb)	AR	Craniofacial abnormalities, joint contracture, hypotonia, and GI/GU problems	*CHST14 *
Arthrochalasis (VIIa/VIIb)	AD	Congenital bilateral hip dislocation	*COL1A1/COL1A2 *
Dermatosparaxis (VIIc)	AR	Sagging skin, delayed fontanels closure, eye lid edema, and short stature and fingers.	*ADAMTS2 *
Periodontal (VIII)	AD	Severe early-onset periodontitis	*12p13 *
Occipital horn syndrome (IX)	XLR	Loose skin, delayed intelligence, hernias, twisted blood vessels, dysautonomia, and hair abnormalities.	*ATP7A *
Spondylocheirodysplastic	AR	Short stature and mild skeletal dysplasia	*SLC39A13 *
EDS-Stickler	AR	Pierre-Robin sequence and eye abnormalities.	*PLOD3 *
EDS-OI	AD	Bone fragility and classic EDS symptoms	*COL1A1/COL1A2 *
Brittle cornea syndrome	AR	Ocular fragility and keratoconus	*ZNF469* *PRDM5 *
Progeroid EDS	AR	Wrinkled face, curly fine hair, and periodontitis	*B4GALT7 *

AR: autosomal recessive; AD: autosomal dominant; XLR: X-linked recessive; OI: osteogenesis imperfecta; GI: gastrointestinal; GU: genitourinary; and ?: unknown.

## Abbreviations

COL: Collagen
EDS: Ehlers -Danlos syndrome
FBN1: Fibrillin 1
OI: Osteogenesis imperfecta
TGF*β*: Transforming growth factor beta
NICU: Neonatal intensive care unit.
